# Supplementary material for: Global Analysis of microRNA-like RNAs Reveals Differential Regulation of Pathogenicity and Development in Fusarium oxysporum HS2 Causing Apple Replant Disease
Source: J Fungi (Basel). 2024 Dec 19;10(12):883. doi: 10.3390/jof10120883 (PMC11676187; doi:10.3390/jof10120883)
Supplement: Supplementary file 1 [file jof-10-00883-s001.zip › jof-3360939-supplementary Figure S1-S3.pdf]

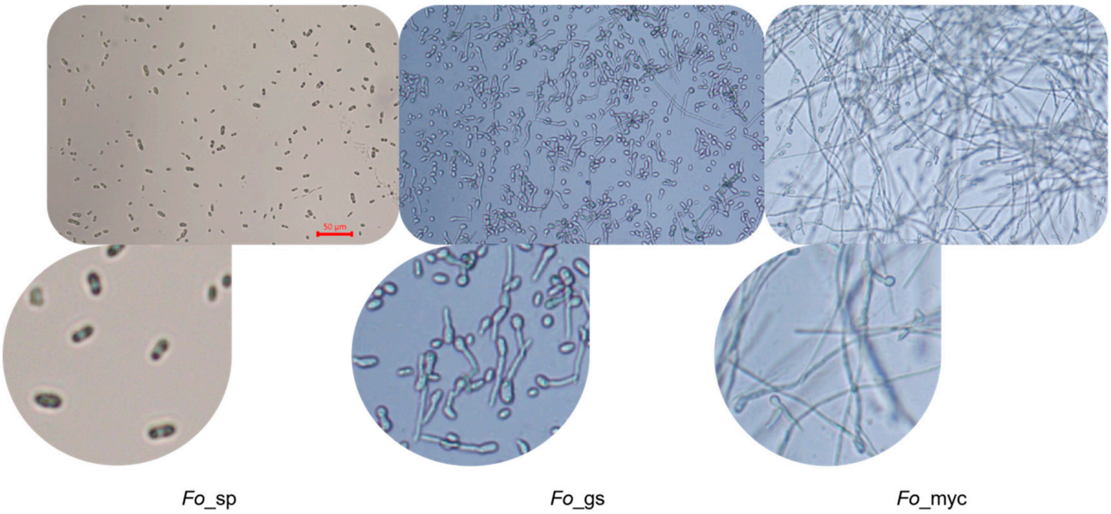


Figure S1 Schematic diagram of samples from three growth stages for *Fusarium oxysporum HS2*.

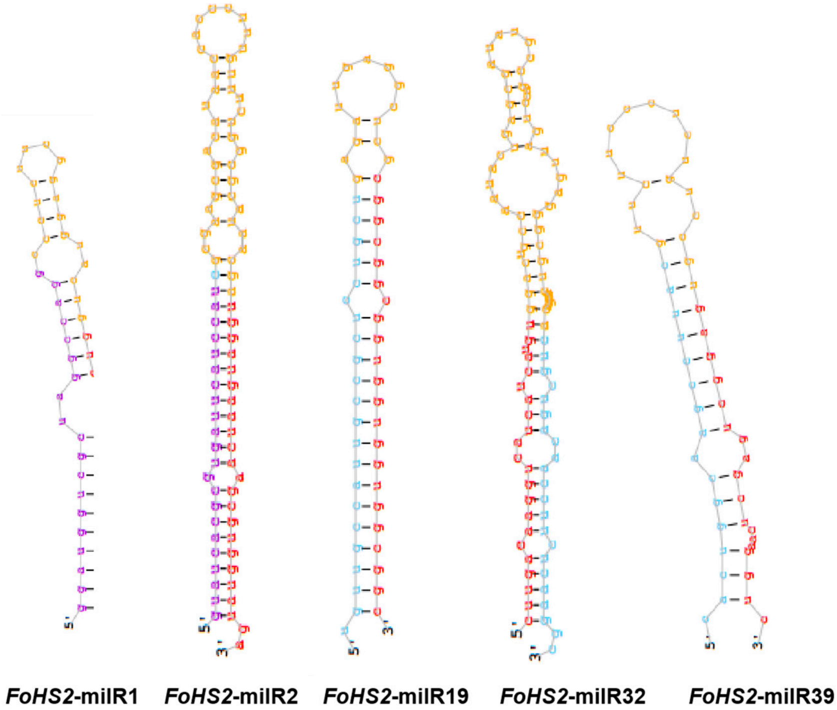


Figure S2 The hairpin structures of core milRNAs precursors at different growt h stages

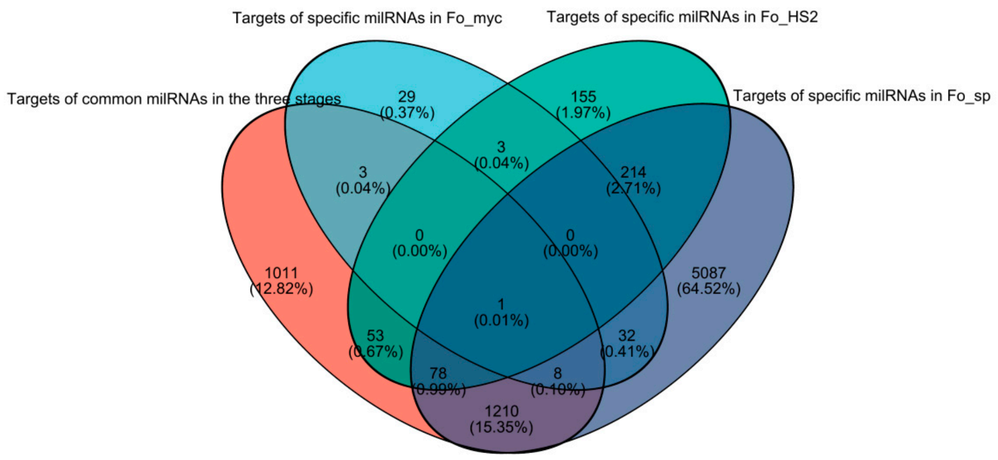


Figure S3 Venn Diagram showing the predicted number of target gene for milRNA at different growth stages
